# Prevalence and Severity of Self‐Assessed Hallux Valgus in Community‐Dwelling Older South African and Flemish Individuals

**DOI:** 10.1002/jfa2.70173

**Published:** 2026-06-02

**Authors:** Marise Carina Breet, Benedicte Vanwanseele, Hylton B. Menz, Tonya M. Esterhuizen, Ranel Venter

**Affiliations:** ^1^ Human Movement Biomechanics Department of Movement Sciences KU Leuven Leuven Belgium; ^2^ Department of Exercise, Sport, and Lifestyle Medicine Faculty of Medicine and Health Sciences Stellenbosch University Stellenbosch South Africa; ^3^ Discipline of Podiatry School of Allied Health Human Services and Sport La Trobe University Melbourne Victoria Australia; ^4^ Division of Epidemiology and Biostatistics Department of Global Health Faculty of Medicine and Health Sciences Stellenbosch University Stellenbosch South Africa

**Keywords:** footwear habits, hallux valgus, older population, prevalence, severity

## Abstract

**Introduction:**

Hallux valgus (HV) is a common foot deformity in older adults, frequently associated with pain and functional impairment. Global prevalence is estimated at 19%, although it varies by continent; consequently, there is a notable knowledge gap concerning its prevalence and severity in older populations of South Africa and Belgium. This study aimed to compare HV prevalence and severity, report on footwear habits within these cohorts, and examine associations with sex, age, and body mass index (BMI).

**Methods:**

This observational cross‐sectional study recruited 300 community‐dwelling adults aged ≥ 60 years from Flanders, Belgium (*n* = 145) and the Western Cape, South Africa (*n* = 155). Demographic data, footwear habits, and HV self‐assessment scores (using the Manchester scale) were collected via an online questionnaire. Associations between HV and other variables were examined using chi‐square tests.

**Results:**

HV prevalence was 27.6% in the Flemish cohort and 31.6% in the South African cohort. Although South Africans reported more frequent barefootedness, HV prevalence rates remained similar. HV prevalence was significantly higher in females (35.4%) compared to males (17.0%). Furthermore, prevalence and severity increased with age but were inversely associated with BMI.

**Conclusion:**

HV prevalence is similar in older South African and Flemish individuals, despite differing footwear habits, supporting the multifactorial nature of HV formation. These findings emphasize the need for region‐specific data and clinical strategies to address HV in older populations.

## 1 Introduction

Hallux valgus (HV) is a common foot deformity frequently observed in older adults. HV is characterized by a three‐dimensional deformity involving the lateral deviation of the great toe, medial deviation of the first metatarsal, and progressive subluxation of the first metatarsophalangeal joint [1]. HV is associated with pain [2], reduced general and foot health‐related quality of life [3], impaired physical function [4], gait instability [5], and an increased risk of falls [6]. Initial HV treatment typically involves non‐surgical approaches, with practitioners primarily relying on their clinical knowledge and expertise due to limited empirical evidence [7]. While surgical intervention may improve pain and provide a slight enhancement in function, these benefits are often overshadowed by potential surgical complications [8]. The pooled recurrence rate of 25% following HV surgery highlights the importance of prevention and early intervention [9].

A 2023 systematic review estimated the global HV prevalence at 19%, with 23% in women and 11% in men [9]. HV prevalence varies by continent, with the highest rates observed in Oceania (29%), Asia (22%), and Europe (18%) and the lowest in Africa (3%) [9]. While studies across regions have demonstrated varying HV prevalence, no peer‐reviewed research has examined HV prevalence and severity in older South African and Flemish populations to date. Understanding the interplay between the intrinsic and extrinsic factors associated with HV development and progression could answer questions regarding these varying prevalence rates and influence the development of effective treatment strategies.

Intrinsic factors, such as female sex [10], genetics [11], and increased age [12], contribute to HV prevalence and severity. However, an increased BMI is inversely associated with HV risk [13, 14, 15]. Extrinsic risk factors include footwear that is too short [16, 17], narrow [12, 18], or has an elevated heel [19]. Early life footwear exposure may influence foot morphology and potentially lead to foot deformities later in life [16, 20, 21, 22, 23]. Limited recent data on shoe‐wearing patterns exist for older populations in South Africa and Flanders [20, 21, 22, 24, 25].

South Africa exhibits a “barefoot culture” with 90.9% of children habitually barefoot and demonstrating differences in foot morphology compared to German children [26]. Research has primarily focused on footwear fit and wearing patterns, and preschool children are often encouraged to attend school barefoot rather than wearing footwear [16, 17, 27]. However, South African school uniform regulations require children to wear footwear based on European data [28]. To the best of our knowledge, no studies have compared HV prevalence and severity and the potential influence of footwear habits in Flanders, Belgium, and Western Cape, South Africa.

This study aimed to compare the prevalence and severity of HV deformity and describe footwear habits among community‐dwelling older adults in Western Cape, South Africa, and Flanders, Belgium. Furthermore, this study examined the associations between age, sex, BMI, and HV prevalence and severity to explore intercontinental differences and factors associated with HV deformities.

## 2 Methods

This observational cross‐sectional study estimated the prevalence and severity of HV in older individuals in Flanders, Belgium, and the Western Cape Metropole, South Africa. A convenience sampling approach recruited community‐dwelling older men and women who voluntarily responded to invitations distributed via flyers, letters, and senior organizations in Leuven, Belgium, and the Western Cape, South Africa. Participants completed an online questionnaire between February 2024 and June 2025. Eligible participants were residents of Flanders, Belgium, or the Western Cape, South Africa, aged ≥ 60 years, and able to stand independently. Individuals with lower limb amputations, blindness, or cognitive impairment that precluded the completion of the questionnaire were excluded. This study was approved by the Health Research Ethics Committee of Stellenbosch University (protocol no. S23/07/150 (PhD)), KU Leuven's Privacy and Ethics platform (PRET), and the Social and Societal Ethics Committee (SMEC) of KU Leuven (protocol no. G‐2024‐7981‐R3 (AMD)). This study was conducted in accordance with the principles of the Declaration of Helsinki.

Data were collected using online questionnaires hosted on the SunSurvey and LimeSurvey platforms. Participants voluntarily accessed the questionnaires via a smartphone using a QR code on flyers, newsletters, or invitation emails from various senior organizations. Questionnaires were available in Afrikaans, English, Xhosa, and Flemish. Participants provided informed consent before completing a questionnaire that recorded their age, weight, height, sex, and footwear habits. BMI classification was based on the optimal functional capacity of older adults, encompassing the following categories: underweight (< 25 kg/m^2^), ideal weight (25–35 kg/m^2^), and overweight (> 35 kg/m^2^) [29]. Footwear habits were recorded using a 10‐item questionnaire adapted from Hollander et al. [30], which assessed the frequency of barefoot versus shod activity throughout the participants' lifespans on a continuous scale. Higher total scores indicated more time spent wearing shoes, whereas lower scores indicated more time spent barefoot.

Hallux valgus self‐assessment was performed using the Manchester scale, which comprises standardized images of the left and right feet. The Manchester scale is a validated, standardized, non‐invasive instrument commonly used for HV deformity classification based on the visual assessment of the external angular alignment relative to the first metatarsal and does not include radiographic or clinical assessment of the joint structure [31]. Self‐assessment using the Manchester scale has demonstrated high test‐retest reliability and acceptable agreement with examiner ratings [32]. Images were categorized as representing no deformity (score = 0), mild deformity (score = 1), moderate deformity (score = 2), or severe deformity (score = 3). Grades 2 and 3 were considered indicative of hallux valgus deformity, with different grades reflecting varying degrees of severity. To analyze HV severity, both feet of each participant were assessed, resulting in nine possible bilateral severity combinations. For participants with asymmetrical severity, the foot with the highest severity was used for the analysis. This approach allowed nine scenarios to be collapsed into four categories (none, mild, moderate, and severe) for descriptive analyses.

The required sample size for estimating prevalence was calculated using a formula for a single population proportion:

$$n=\frac{Z^{2}\;x\;p\left(1-p\right)}{d^{2}}.$$

A *Z*‐value of 1.96 was used for a 95% confidence interval (*α* = 0.05). Prevalence estimates were derived from previous literature, which reported an overall HV risk of 13% in males and 30% in older women. Accordingly, proportions of 0.13 (males) and 0.30 (females) were used [33]. The *d* value was set at 0.05, corresponding to a total confidence interval width of 10%, as recommended by Charan and Biswas (2013) [34]. For between‐population comparisons, a sample size of 200 participants per group was estimated to provide 80% power to detect a 10% difference in proportions at a two‐sided significance level of 0.05. Owing to recruitment constraints, the final sample comprised 145 Flemish and 155 South African participants, which was slightly fewer than our original sample size estimation.

Data were analyzed using IBM SPSS Statistics software (version 30.0). Continuous variables are presented as means and standard deviations, and categorical variables as percentages. Associations between HV prevalence and age group, sex, and BMI category were examined using chi‐square tests. Between‐cohort differences in HV prevalence, severity, footwear habits, and associated factors were assessed by stratified analyses using chi‐squared tests for categorical variables and independent‐samples t‐tests for continuous variables. Statistical significance was set at *p* < 0.05.

## 3 Results

This study included 300 participants, comprising 94 men and 206 women. Table 1 summarizes the characteristics of the study population. Footwear habit scores represent the average time spent barefoot or shod throughout an individual's lifespan, with higher scores indicating a greater time spent wearing footwear. HV prevalence refers to the incidence of HV on either the left or right foot, graded as moderate (grade 2) or severe (grade 3). As shown in Table 1, the HV prevalence in Flanders, Belgium and the Western Cape, South Africa cohorts was 27.6% and 31.6%, respectively, with similar percentages observed for mild, moderate, and severe cases. There was no statistical difference between the cohorts in terms of prevalence or severity (*p* = 0.445 and *p* = 0.244).

**TABLE 1:** Demographics, HV prevalence and HV severity according to cohort.

		Flemish *n* = 145	South African *n* = 155
Sex *n* (%)	Male	57 (39.3)	37 (23.9)
	Female	88 (60.7)	118 (76.1)
Age mean (SD), *y*		71.4 (7.4)	72.7 (8.0)
BMI mean (SD), kg/m^2^		25.4 (4.2)	27.7 (5.1)
Foot dominance *n* (%)	Right	106 (73.1)	124 (80.0)
	Left	39 (26.9)	31 (20)
Footwear habits score out of 10 median (IQR)		10 (10–10)	8 (6–9)
HV prevalence It	Left and right foot	40 (27.6) [95% CI 20.7%–35.7%]	49 (31.6) [95% CI 24.5%–39.6%]
HV severity *n* (%)	None (Grade 0)	46 (31.7)	47 (30.3)
	Mild (Grade 1)	59 (40.7)	59 (38.2)
	Moderate (Grade 2)	24 (16.6)	35 (22.5)
	Severe (Grade 3)	16 (11.0)	14 (9.0)

**FIGURE 1:**
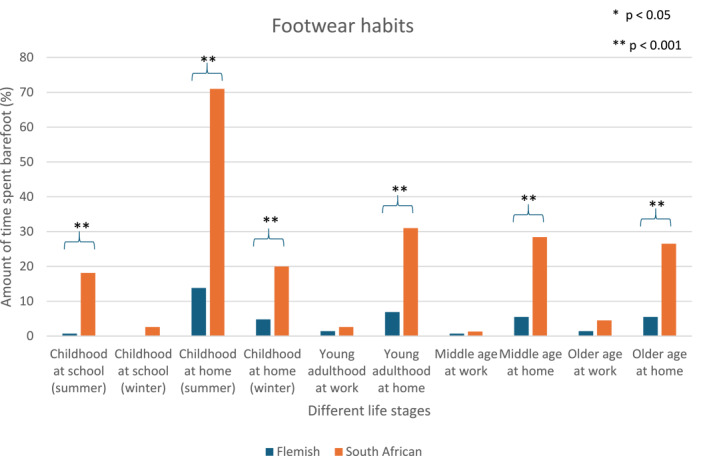
Distribution of barefootedness across life stages of both cohorts based on the barefoot questionnaire.

Figure 1 shows that footwear habits differ significantly between cohorts across life stages. The Flemish cohort spent less time barefoot, whereas the South African cohort spent more time barefoot during childhood, young adulthood, middle age, and old age, particularly in settings where barefoot walking is customary, such as at home.

### 3.1 Factors Associated With HV Prevalence

**FIGURE 2:**
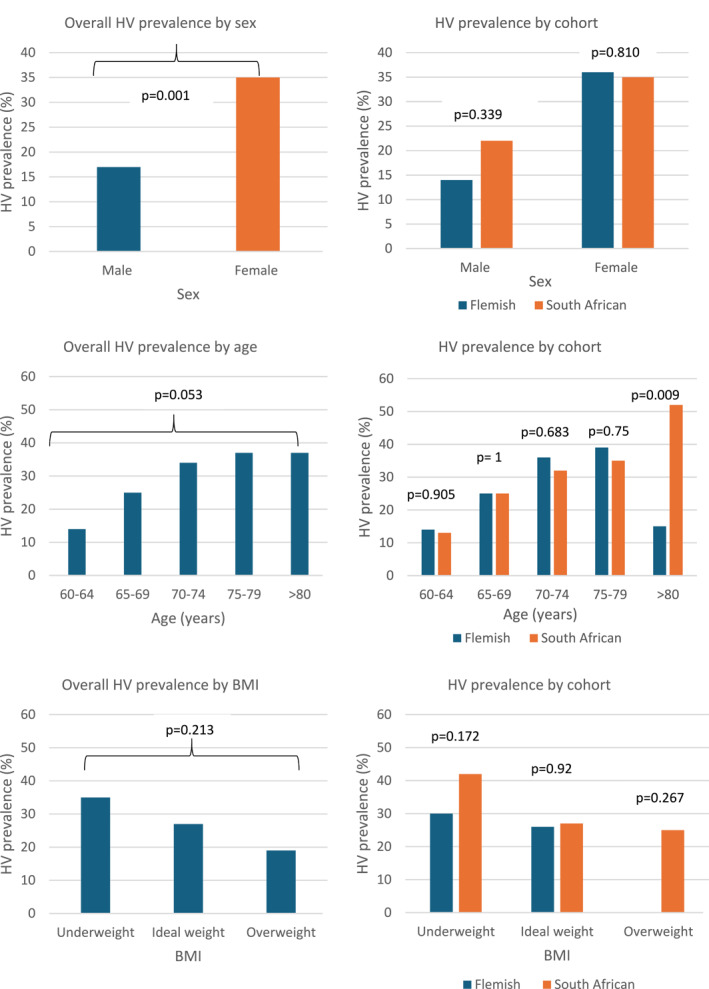
HV prevalence according to sex, age, and BMI.

The associations between HV prevalence by cohort and sex, age, and BMI are shown in Figure 2. As shown in Figure 2, sex differences significantly affected HV prevalence (*p* = 0.001) in the combined Flemish and South African cohorts. Overall, males had an HV prevalence of 17%, whereas females had a prevalence of 35%. Within male and female participants, there were no differences between the cohorts in terms of HV prevalence (*p* = 0.339 and 0.810, respectively). There was no overall association between age group and HV prevalence in the combined cohorts (*p* = 0.053). When stratified by age group, a significant difference (*p* = 0.009) in HV deformity prevalence was observed only in participants aged > 80 years between the Flemish (15%) and South African (52%) cohorts. HV prevalence declined non‐significantly with increasing BMI (*p* = 0.213) overall, and there was no difference between cohorts within BMI categories, although the overweight Flemish cohort had no HV cases.

### 3.2 Factors Associated With HV Severity

**FIGURE 3:**
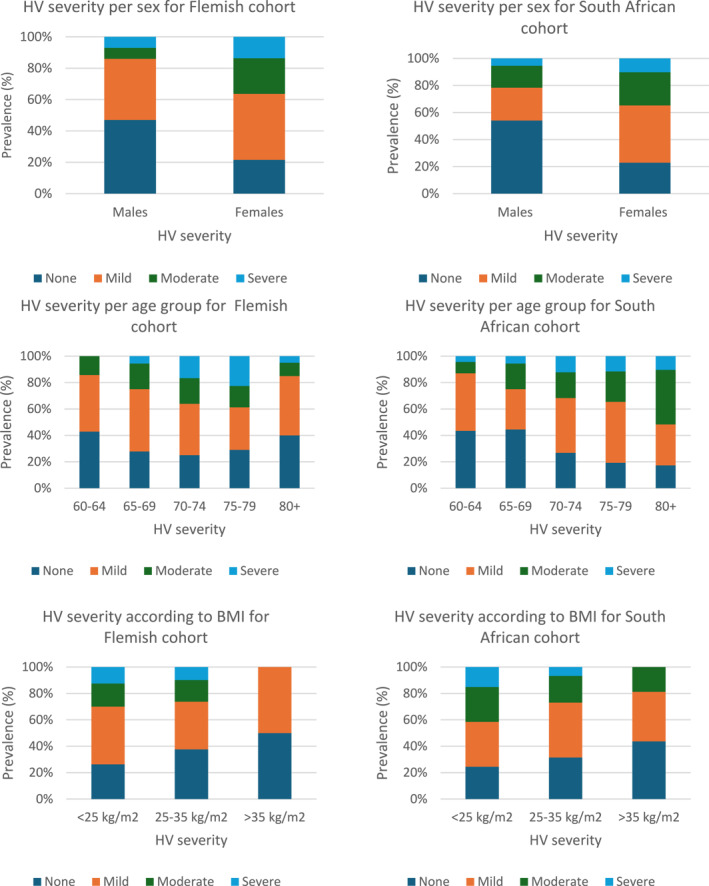
Prevalence of HV severity per cohort according to sex, age, and BMI.

HV severity categories of none (grade 0), mild (grade 1), moderate (grade 2), and severe (grade 3) exhibited similar percentages in both cohorts (Table 1). The prevalence of HV severity stratified by sex, age, and body mass index is illustrated in Figure 3. Both the Flemish and South African men had the highest prevalence of no HV, at 47% and 54%, respectively. Conversely, Flemish and South African women exhibited the highest prevalence of mild HV (42%). Furthermore, females from both cohorts had a higher prevalence of moderate and severe HV than males.

Age groups 60–64 and 65–69 years presented with a high prevalence of no or mild HV cases in both cohorts. An increase in HV severity to moderate and severe HV was correlated with increasing age for both cohorts, except for the Flemish 80+ age group, which presented with a lower prevalence of moderate and severe HV.

In both cohorts, underweight participants exhibited the highest prevalence of moderate and severe HV, whereas overweight participants demonstrated the highest percentages of no and mild HV. South Africans reported the highest prevalence of mild HV cases in the ideal weight category. Notably, no moderate or severe HV cases occurred in the overweight group from the Flemish cohort, and no severe HV cases were identified in the South African cohort.

## 4 Discussion

To our knowledge, this is the first study that compared HV prevalence, HV severity, and footwear habits among community‐dwelling older men and women from the Western Cape, South Africa, and Flanders, Belgium, and investigated their associations with sex, age, and BMI. The HV prevalence and severity in both cohorts differed from previously reported percentages in Europe and Africa [9]. Footwear habits varied significantly across cohorts; South African participants reported spending more time barefoot during childhood, young adulthood, middle age, and old age than Flemish participants. An unexpected finding was that, despite more barefoot exposure in the South African cohort, HV prevalence remained similar despite Flemish adults spending more time in footwear throughout their lives, which contrasts with previous research [21, 22, 23]. HV prevalence and severity were higher in women and older individuals. Consistent with previous studies, BMI had an inverse effect on HV prevalence and severity [14].

The Flemish and South African cohorts showed HV prevalence rates of 27.6% and 31.6%, respectively, compared to a global prevalence of 19% [9]. The pooled global prevalence of 35.7% in individuals aged ≥ 60 years is consistent with these findings [33]. Country‐specific data indicated that Europe accounted for 18.35% and Africa for 3% in HV prevalence [9]. To our knowledge, no region‐specific research has examined HV prevalence in Flanders, Belgium, or the Western Cape, South Africa. The prevalence of HV in Flanders exceeds the pooled European prevalence, while the African data contrast significantly with the South African findings. This discrepancy may be attributable to the underrepresentation of HV prevalence data in certain European and African regions. Recent research reported an HV prevalence of 13.6% in Nigeria, which is lower than that observed in the South African cohort. The HV rates in Flanders and South Africa align with those from Japan, Spain, Pakistan, India, and Saudi Arabia, showing a 25%–43% prevalence [13, 35, 36, 37, 38].

The available literature on HV severity is limited. HV severity scores for moderate and severe HV in both cohorts matched those of Japanese residents in a mountain village, except for mild HV at 66.3% in the Japanese cohort and 40.7% and 38.2% in the Flemish and South African cohorts [35]. Both studies had predominantly female participants; however, the Japanese study also included individuals with knee osteoarthritis, a condition associated with increased HV severity [35].

In South Africa, 90.9% of children and adolescents are reported to be habitually barefoot [26]. The older South African cohort showed a similar lifelong barefoot tendency, in contrast to the Flemish population. Previous studies have reported associations between footwear habits and HV prevalence, with a higher HV prevalence observed in populations with prolonged footwear use and structural adaptations linked to the frequency of footwear exposure [20, 21, 22]. Based on this literature, it can be hypothesized that greater exposure to barefoot conditions may be associated with a lower prevalence of HV deformity; however, similar HV prevalence rates were observed in the Flemish and South African cohorts. Habitually barefoot populations demonstrate differences in foot morphology, including foot length, width, and dynamic arch indexes, compared to predominantly shod populations [26, 39]. Although footwear design in South Africa has been reported to rely on European datasets [28], it has been suggested that when footwear is worn, mismatches between foot morphology and footwear may occur. However, this study did not assess footwear fit, and no conclusions can be drawn regarding its contribution. Previous studies have reported associations between HV deformity and footwear characteristics, such as narrow toe boxes and elevated heels [14, 17, 19, 37, 40, 41]; however, these factors were not evaluated in the present study.

Sex significantly influenced HV prevalence and severity in both cohorts, with females demonstrating higher percentages of mild and moderate HV than males. This finding is consistent with previous studies reporting a higher HV deformity among women [9, 13, 14, 35, 36, 38]. While prior research has suggested associations with the aforementioned footwear characteristics, as well as anatomical and hormonal factors [42], the current study did not assess footwear characteristics and therefore cannot determine their contribution.

HV prevalence and severity increased with age, consistent with previous studies [9, 12, 14, 37, 38]. The cohorts exhibited differences in peak age: South Africans demonstrated the highest prevalence and moderate HV severity in the 80+ age group, while the Flemish cohort peaked in the 75–79 age group with severe HV deformity. The prevalence of HV has previously been linked to increased age, which supports the increase in HV prevalence in the 80+ age group [9, 33]; however, other studies have also reported peaks in HV prevalence as early as 61–70 years of age [43]. The reasons for these discrepancies remain unclear and may be related to population‐specific factors or sampling variations, including potential selection bias in older age groups. These differences could also be related to variations in the individuals studied or exposure to different footwear conditions during early life.

Prevalence was inversely associated with increasing BMI categories for both males and females, consistent with the findings reported by Dufour et al. [14], who also reported a negative association between HV prevalence and BMI for both sexes. Participants in the underweight and ideal BMI categories exhibited the highest prevalence and greater percentages of moderate and severe HV deformity, whereas those in the overweight category demonstrated the lowest prevalence and highest percentage of no or mild HV deformity. The reason for this inverse association remains unclear. Previous studies have reported inconsistent associations between BMI and HV, suggesting that BMI is likely a complex and context‐dependent factor in HV development, with no consistently established single mechanism. Furthermore, no sex‐specific pattern was observed in the association between hallux valgus and BMI in this study. This contrasts with the findings of Nguyen et al. [15], who reported sex‐specific associations. This aligns with the broader heterogeneity in literature.

### 4.1 Significance

This study provides updated prevalence data for older adults and highlights the associations with age, sex, and body mass index. Given the high recurrence rate of HV following surgery, these findings could aid in risk assessment and patient education and ultimately address the burden of HV in older populations. While this study contributes to the limited research comparing intercontinental footwear habits between older South African and Flemish populations, being habitually barefoot did not appear to have a protective effect against the development of HV deformity. Further research incorporating a detailed assessment of footwear characteristics is needed to understand these relationships. Finally, this study found that these cohorts differ from previously reported continental averages, suggesting that HV prevalence data may be underrepresented within continents and highlighting the need for region‐specific studies.

### 4.2 Limitations

This study had several limitations that should be considered when interpreting the findings. A limitation of this study is the use of self‐assessed HV classification using the Manchester scale. This tool has demonstrated good reliability and validity and is widely used in epidemiological research. It offers several important advantages, including being non‐invasive, avoiding exposure to ionizing radiation, and enabling standardized assessment and comparison across large populations. However, although the validity of the Manchester Scale has been demonstrated, the reliability of participant self‐assessment has not yet been fully established. Future studies should further evaluate the reliability of the Manchester Scale when used for self‐assessment.

In addition, the Manchester scale is based on visual assessment of forefoot morphology and does not include radiographic or clinical confirmation. It may therefore not distinguish hallux valgus from hallux rigidus, which can present similarly in older adults, introducing potential misclassification and residual confounding. HV prevalence estimates therefore reflect deformity classification rather than clinically or radiographically confirmed diagnosis and should be interpreted accordingly.

Second, the cohorts were compared according to HV severity by including both feet of each participant. Nine bilateral severity scenarios were created, resulting in some groups being small and limiting the ability to perform meaningful statistical comparisons. These groups were combined into four categories: none, mild, moderate, and severe. Consequently, factors potentially impacting HV severity were reported descriptively rather than tested inferentially.

Third, engaging with older participants presented inherent limitations, such as retrospective reporting of footwear habits, particularly from their younger years. Due to the long recall period, exposure misclassification is likely, with a probable reduced accuracy of footwear use. However, as this limitation applied equally across the study groups, it is unlikely to have introduced a systematic bias between cohorts but may have reduced the observed associations between footwear exposure and HV. Despite this, the findings align with previous South African studies on the footwear habits of South African and German children. Additionally, challenges arose concerning the technological skills required to complete an online questionnaire. Although printed copies were made available, they were seldom requested, and incomplete responses could not be pursued further because of the anonymous and voluntary nature of the study design.

Fourth, the flyers mentioned the aim to investigate risk factors for developing foot deformities, which could make those with deformities more likely to respond, and therefore create a selection bias. Fifth, the cross‐sectional design of this study allowed the identification of associations between HV and factors such as age, sex, and BMI; however, it did not permit conclusions about causality. Sixth, we used a convenience sample, which limits the generalizability of the findings to other populations or to the broader national context. Finally, although a priori calculations suggested that 200 participants per cohort were required to detect a 10% difference in prevalence with 80% power, the achieved sample size was smaller than anticipated. While the cohorts were relatively balanced, certain subgroups contained limited numbers, particularly South African males with moderate and severe HV. Similar constraints were observed in smaller age groups with moderate and severe HV, notably the Flemish 60–64 and 80+ groups. These underpowered subgroups may have reduced statistical power and estimate precision, potentially limiting the ability to detect small between‐group differences and warranting cautious interpretation of these findings.

## 5 Conclusion

To our knowledge, this study represents the first comparative analysis of HV prevalence and severity between older adults from Flanders, Belgium, and the Western Cape, South Africa. Despite significant differences in lifelong footwear habits, with the South African cohorts reporting greater frequency of barefoot walking, both groups exhibited similar HV prevalence and severity. These findings reinforce the multifactorial nature of HV, confirming that increased prevalence and severity are associated with advancing age, female sex, and lower or ideal body mass index. This study underscores the importance of considering age, sex, BMI, and cumulative footwear exposure to address the burden of HV in older populations.

## Author Contributions

**Marise Carina Breet:** writing – original draft, writing – review and editing, conceptualization, data curation, formal analysis, investigation, methodology, project administration, resources, validation, visualization. **Benedicte Vanwanseele:** conceptualization, methodology, supervision, validation, visualization, writing – review and editing. **Hylton B. Menz:** conceptualization, supervision, validation, visualization, writing – review and editing. **Tonya M. Esterhuizen:** formal analysis, validation, writing – review and editing. **Ranel Venter:** conceptualization, methodology, supervision, validation, visualization, writing – review and editing.

## Funding

The authors have nothing to report.

## Ethics Statement

This study received approval from the Health Research Ethics Committee at Stellenbosch University (protocol no: S23/07/150 (PhD)), KU Leuven's Privacy and Ethics platform (PRET), and the Social and Societal Ethics Committee (SMEC) of KU Leuven (protocol no: G‐2024‐7981‐R3 (AMD)). The study adhered to the Declaration of Helsinki principles. All participants provided written informed consent before their participation.

## Consent

All participants provided written informed consent to the publication of the results.

## Conflicts of Interest

The authors declare no conflicts of interest.

## Data Availability

Data supporting the findings of this study are available upon reasonable request.
